# Neurocysticercosis Presenting With a New-Onset Seizure: A Case Report

**DOI:** 10.7759/cureus.13897

**Published:** 2021-03-15

**Authors:** William Lim, Muhammed Atere, Bryan Nugent, Swann Tin, Ambreen Khalil

**Affiliations:** 1 Internal Medicine, Richmond University Medical Center, Staten Island, USA; 2 Medicine, Richmond University Medical Center, Staten Island, USA; 3 Internal Medicine/Infectious Disease, Richmond University Medical Center, Staten Island, USA

**Keywords:** infectious and parasitic diseases, neurocysticercosis, seizure

## Abstract

Neurocysticercosis (NCC) is a common infection that is found worldwide but is often neglected in the United States (US). This case report aims to illustrate the presentation of the disease, provide information on this globally prevalent pathogen, and shed light on the diagnostic workup and treatment of the infection. We discuss the case of a 31-year-old male patient of Central American origin presenting with a new-onset seizure. He had no significant past medical history and had never experienced similar events before. The diagnosis was made through neuroimaging, serum antibody testing, and biopsy of the brain lesion. This case highlights the importance of performing a good clinical history and a proper diagnostic workup that would help in the prompt recognization and treatment of this common worldwide illness that may not be endemic to the clinician’s geographical area.

## Introduction

Cysticercosis is a common infection affecting approximately 50 million people worldwide and endemic to many regions of Central and South America, sub-Saharan Africa, India, and other parts of Asia [1-7]. Neurocysticercosis (NCC) is the most frequent preventable cause of epilepsy worldwide and is estimated to be the cause of 30% of epilepsy cases in endemic areas [8]. However, it is one of the five neglected parasitic infections (NPIs) in the United States (US), targeted by the Centers for Disease Control and Prevention (CDC) for public health action [9]. Due to increased globalization, NCC has become a notable emerging infection in the US. More than 2,300 patients are admitted due to cysticercosis annually in the US, and about 2% of seizure cases admitted to emergency departments are found to be attributed to NCC [10,11].

## Case presentation

**Figure 1:**
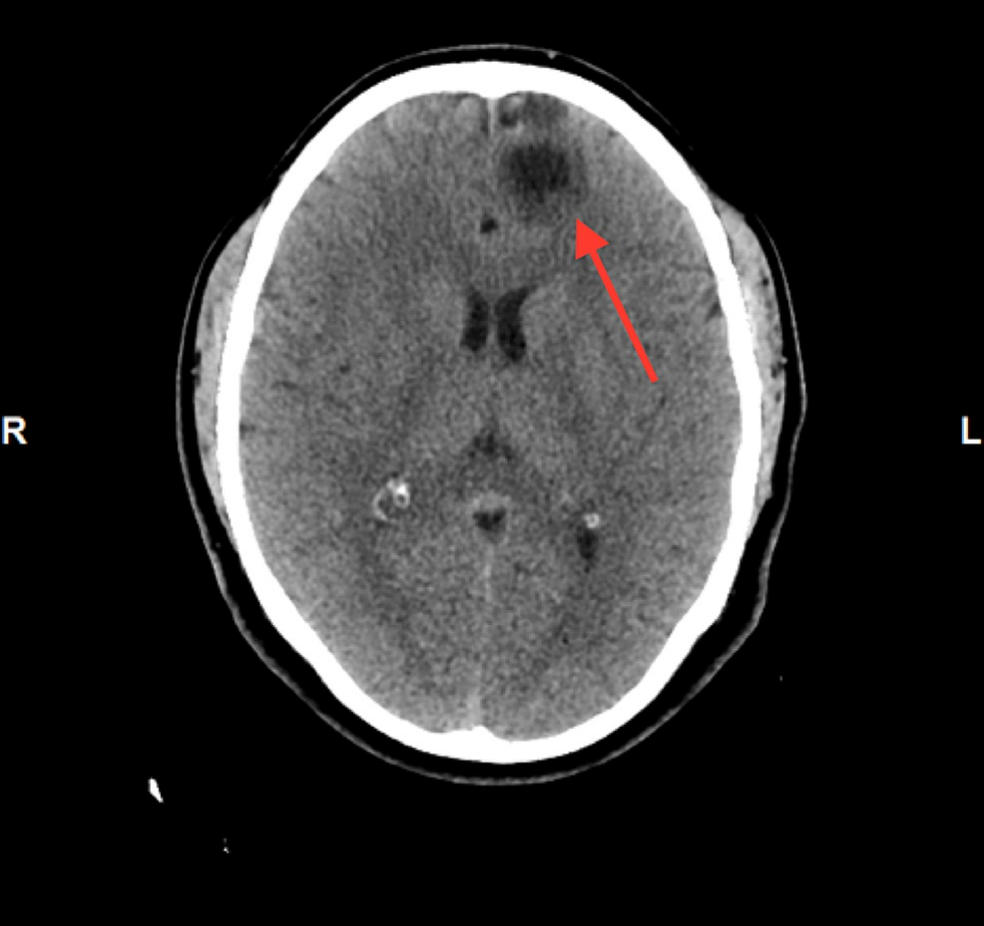
CT head showing a calcified cystic lesion in the anteromedial left frontal lobe CT: computed tomography

**Figure 2:**
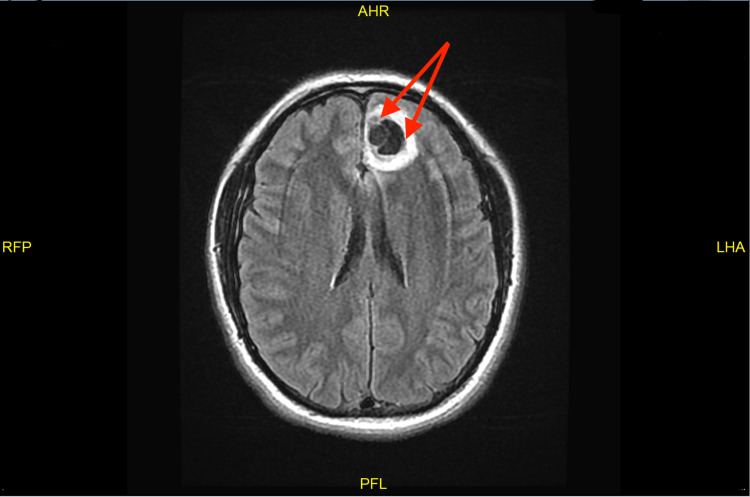
MRI head showing a bilobed cystic mass in the anteromedial left frontal lobe MRI: magnetic resonance imaging

**Figure 3:**
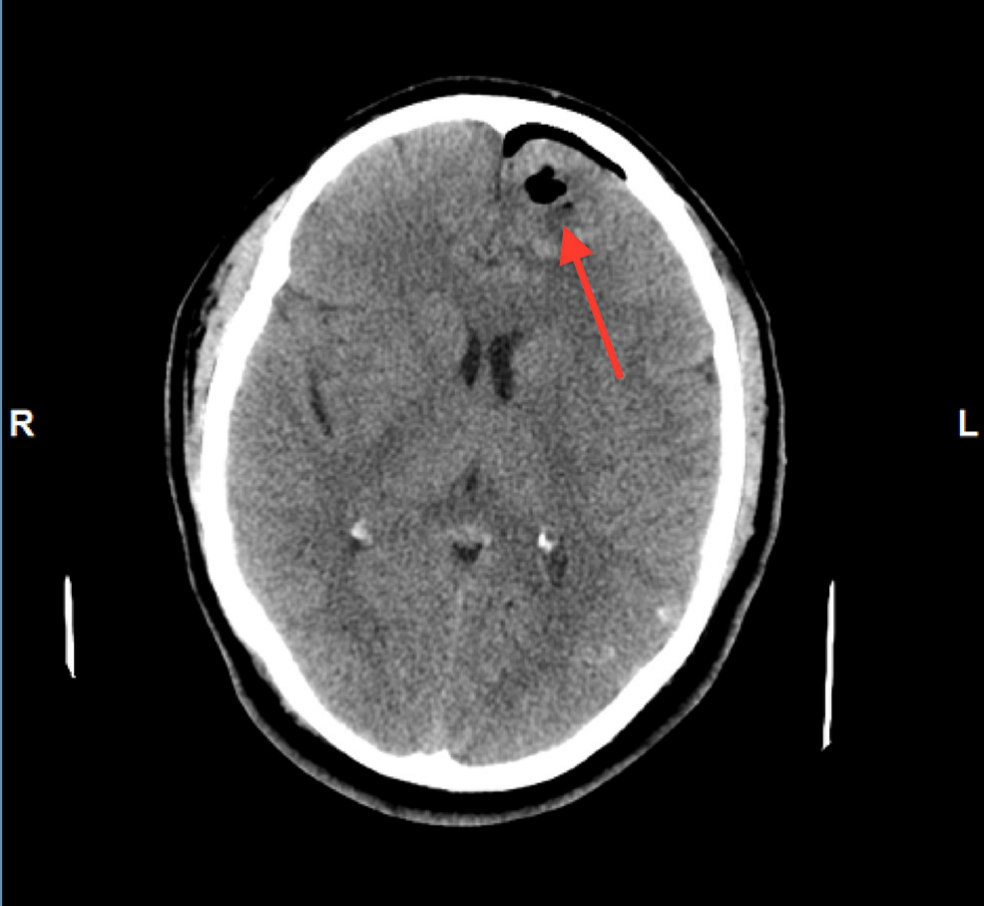
CT head after excisional biopsy CT: computed tomography

**Figure 4:**
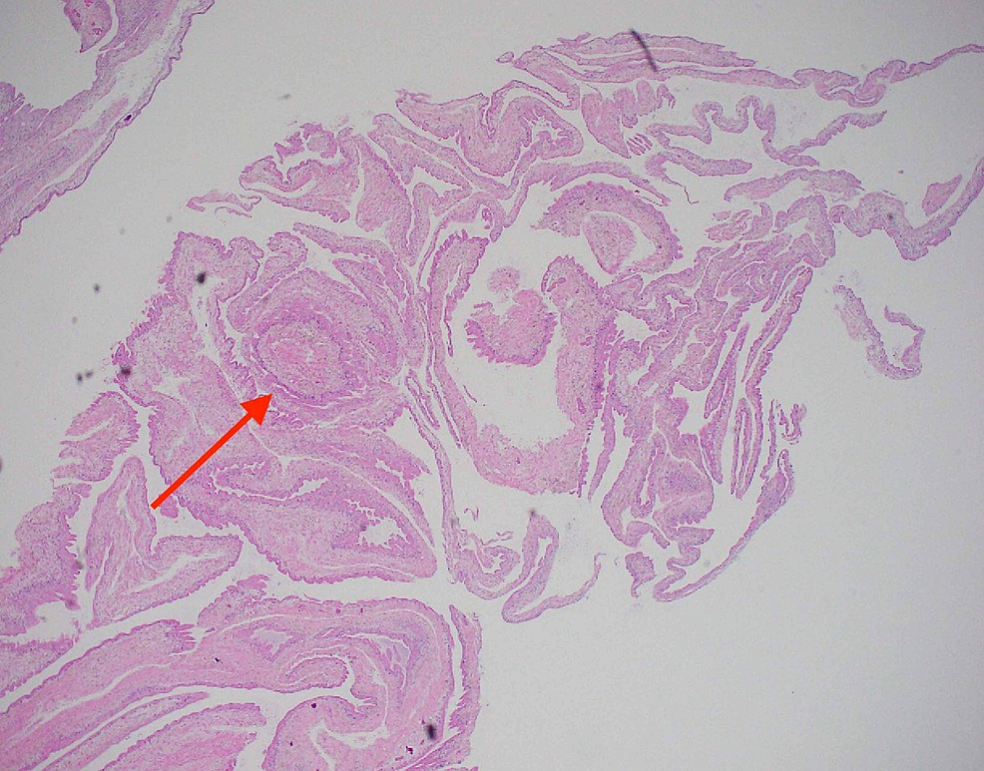
Hematoxylin-eosin stain of cyst biopsy showing lymphoplasmacytic histiocytic inflammation on 40x magnification

**Figure 5:**
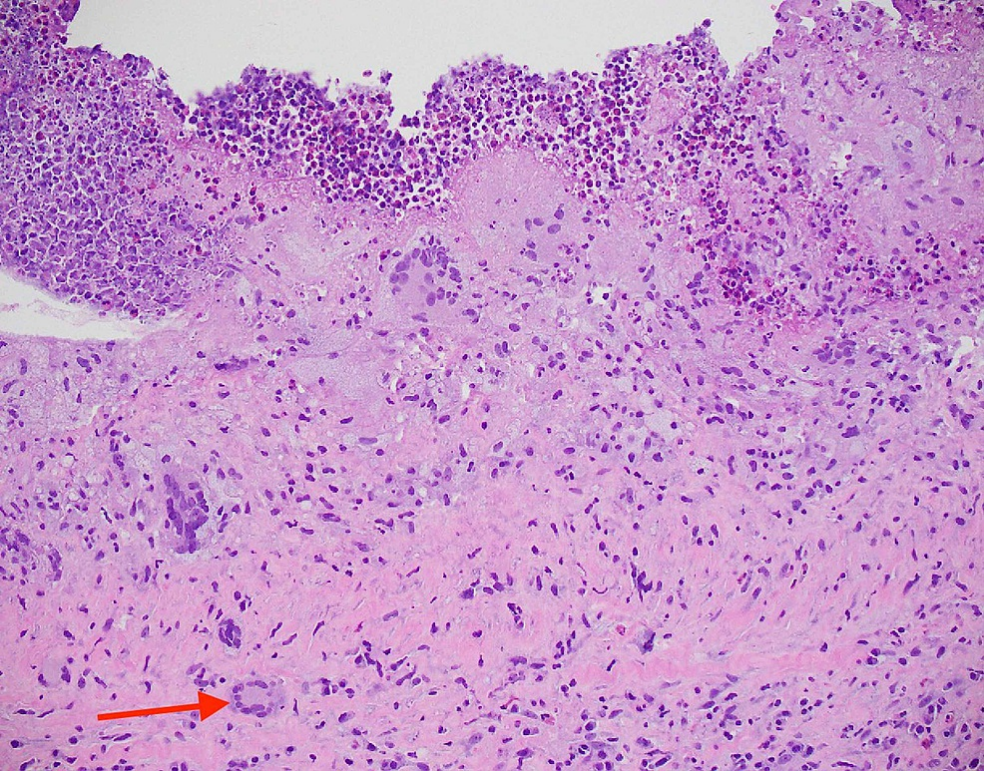
Hematoxylin-eosin stain of cyst biopsy showing foreign-body multinucleated giant cell on 200x magnification

**Figure 6:**
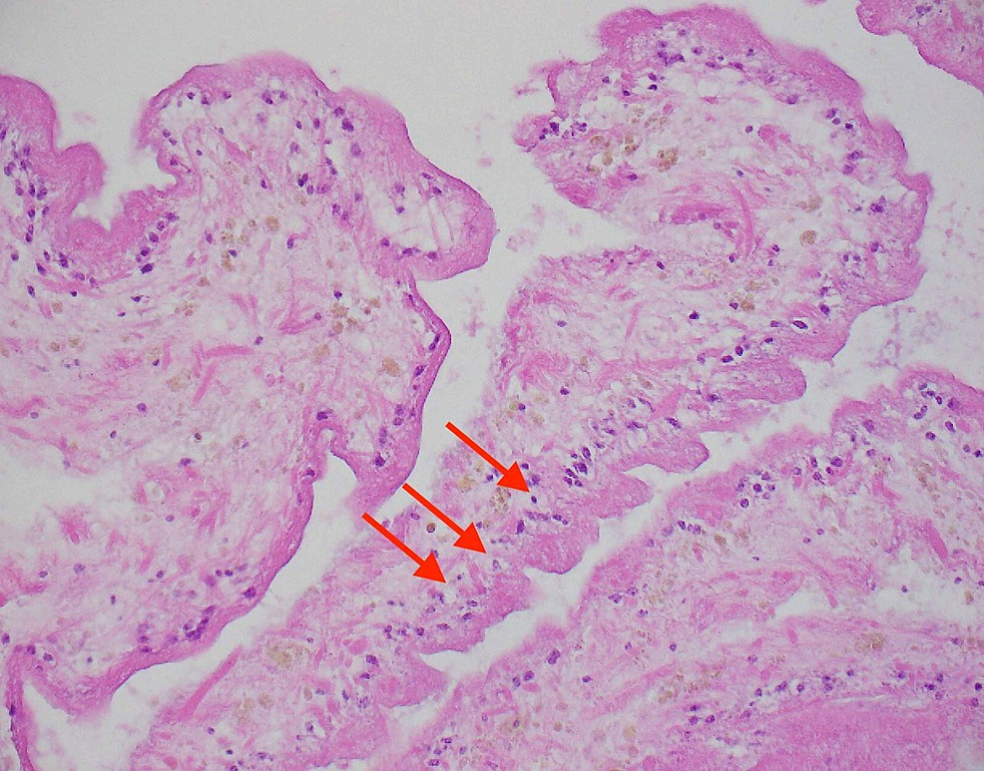
Hematoxylin-eosin stain of cyst biopsy showing reactive gliosis on 200x magnification

A 31-year-old man with no significant past medical history presented to the emergency department with a new-onset seizure. A family member had noticed shaking movement of arms and legs that had lasted for three minutes while the patient had been taking a nap, followed by lethargy and confusion afterward. He denied fever, chills, night sweats, weakness, motor or sensory deficits, and vision changes or recent trauma. The patient had emigrated from Guatemala to the United States two years ago. There were no neurological deficits found on examination. Complete blood count (CBC) showed leukocytosis of 12.8k/UL, with a neutrophilic predominance of 83.3% with no eosinophilia. The urine drug screen was negative. CT scan showed a complex, 3 x 2 cm partially calcified cystic lesion in the anteromedial left frontal lobe (Figure 1). Furthermore, the MRI head revealed a bilobed cystic mass in the anteromedial left frontal lobe (Figure 2). Electroencephalogram (EEG) showed no significant abnormalities. Malignancy was initially suspected as well and the patient underwent craniotomy and excisional biopsy. A repeat CT scan performed after the excisional biopsy can be seen in Figure 3. The pathology did not reveal any malignancy. Instead, it confirmed cysticercosis. Biopsy pictures can be seen in Figures 4-6. Cysticercus antibody was found to be positive, and serology tests that were taken in order to differentiate from other cyst-producing parasitic infections such as Histoplasma antigen and Entamoeba histolytica antibody came back negative as well. Ophthalmology was also consulted, but no ocular involvement was found. Given that the cysts were calcified and there was no ocular involvement such as uveitis, the antiparasitic treatment was deferred and the patient was treated with levetiracetam 500 mg twice a day and discharged with neurosurgery and infectious disease outpatient appointment.

## Discussion

NCC is the most common parasitic infection of the brain, and it is transmitted by the ingestion of Taenia solium eggs shed in the stool of a human tapeworm carrier. Taenia solium can cause both taeniasis (infection with adult tapeworm) and cysticercosis (infection with cysts). A common misconception is that humans can acquire NCC by consuming undercooked pork. Consumption of undercooked pork will only lead to taeniasis because infected pork contains the larval cysts that develop into adult worms in the human intestine, but does not contain the eggs that cause cysticercosis [12]. The recognition of NCC in the acute setting can be challenging in countries outside of the endemic areas since there is no specific diagnostic finding on routine blood work, including peripheral eosinophilia. Stool examination is also insensitive because several years pass between exposure to Taenia solium eggs and the onset of clinical presentation. The approach to diagnosis is based on clinical manifestations, neuroimaging findings, and epidemiologic exposure. The most common clinical manifestation of NCC is a seizure and/or headache. Patients can also present with confusion, vision changes, focal neurologic signs, stroke, and meningitis. Fever is typically absent [13].

The appearance of parenchymal cysts on imaging progresses from non-enhancing (viable phase) to ring-enhancing (degenerating phase) to calcified nodule (non-viable phase) or complete resolution. These forms may coexist simultaneously [13]. CT scan is useful for identifying parenchymal calcifications while MRI is preferred for temporal and frontal lobe lesions close to the skull base, intraventricular cysts, and subarachnoid cyst. Cystic lesions with an eccentric ‘dot’ representing the scolex are pathognomonic findings of NCC. If radiographic appearance is nonspecific, there may be diagnostic difficulties in differentiating NCC from other lesions of the brain such as tuberculomas, other parasitic cysts such as hydatid cysts, and neoplastic lesions such as gliomas and metastatic lesions [14]. Since imaging showed cystic lesions in our patient, both Histoplasma antigen and Entamoeba histolytica antibody were taken alongside Cysticercus antigen in order to rule out other cyst-producing parasitic infections.

As of now, enzyme-linked immunoelectrotransfer blot (EITB) is the best serological test available for the diagnosis of NCC. It has a sensitivity of 98% and specificity of 100% in patients with more than one live cyst or subarachnoid disease, and approximately 60-70% in patients with only one degenerating cyst or calcified lesions only [15,16]. However, it only detects the presence of antibodies in the larval antigen and does not necessarily indicate the presence of active disease. It may be positive in persons with exposure to the parasitic antigen and patients with extraneural cysticercosis. It can also be positive in patients who have been treated for cysticercosis in the past because antibodies may persist for up to one year after successful treatment. On the other hand, negative serology test results do not exclude the diagnosis of NCC in patients with compatible clinical manifestations and radiographic findings [14].

EEG was done in this patient to rule out any other seizure disorder, which showed no significant abnormalities. Brain biopsy is indicated when there is ambiguity in diagnosis [13]. In our case, after discussing with the neurosurgery team and infectious disease team, a decision was made to obtain a biopsy given that the sensitivity of the serology test was poor in calcified lesions and other differential diagnoses such as malignancy or brain abscess could not be ruled out.

Anticonvulsant drug therapy is recommended in patients who present with seizures, even of a single episode, because NCC lesion(s) serve as a nidus for recurrent seizures. Anti-seizure drugs such as phenytoin, carbamazepine, or levetiracetam can be used. Indications for anti-parasitic therapy are confined to patients with viable and/or degenerating cysts but should be done under close supervision as clinically the patient may get worse initially. Anti-parasitic therapy is not recommended in patients with untreated hydrocephalus, high cyst burden, and presence of a calcified lesion(s) only, and hence our patient was not given antiparasitic therapy. When indicated, albendazole (15 mg/kg/day) is used as the first line. In cases where there are more than two cysts, dual therapy with albendazole and praziquantel (50 mg/kg/day) is recommended. The duration of antiparasitic therapy for the treatment of parenchymal NCC is 10-14 days. Adjunctive corticosteroid therapy is recommended to reduce seizures caused by degeneration of viable cysts due to anti-parasitic drugs. The most commonly used regimens are prednisone (1 mg/kg/day) or dexamethasone (0.1 mg/kg/day), and it is recommended that they are started at least one day prior to initiating antiparasitic therapy and should continue throughout the duration of therapy and are tapered over a few days [17,18].

## Conclusions

Our patient presented to the emergency department with a seizure and was found to have NCC, which is a rare cause of seizure in the US. Since there has been an increase in cases of NCC in the US, emergency physicians should consider this in the differential diagnosis of patients presenting with seizures, based on the history of the patient. A multidisciplinary approach is needed to reach a diagnosis in such cases since the presentation of the disease is pleomorphic, requiring a combination of clinical evaluation, imaging, and laboratory investigations.
